# Alterations in cerebral glucose metabolism measured by FDG PET in subjects performing a meditation practice based on clitoral stimulation

**DOI:** 10.12688/f1000research.122351.1

**Published:** 2022-09-07

**Authors:** Andrew B. Newberg, Nancy A. Wintering, Chloe Hriso, Faezeh Vedaei, Feroze B. Mohamed, Sara E. Gottfried, Reneita Ross

**Affiliations:** 1 Department of Integrative Medicine and Nutritional Sciences, Thomas Jefferson University, Philadelphia, PA, 19107, USA; 2 Department of Radiology, Thomas Jefferson University, Philadelphia, PA, 19107, USA; 3 Department of Obstetrics and Gynecology, Thomas Jefferson University, Philadelphia, PA, 19107, USA

**Keywords:** FDG PET, Brain, Meditation, Spirituality, Cerebral Glucose Metabolism, Clitoris, Stimulation

## Abstract

**Background:** The relationship between sexuality, or the libido, and spirituality or religion has long been debated in psychiatry. Recent studies have explored the neurophysiology of both sexual experiences and spiritual practices such as meditation or prayer. In the present study, we report changes in cerebral glucose metabolism in a unique meditation practice augmented by clitoral stimulation called, Orgasmic Meditation, in which a spiritual state is described to be attained by both male and female participants engaged in the practice as a pair.

**Methods:** Male (N=20) and female (N=20) subjects had an intravenous catheter connected to a bag of normal saline inserted prior to the practice. During the practice, men stimulated their partner’s clitoris for exactly 15 minutes (he received no sexual stimulation). Midway through the practice, researchers injected 18F-fluorodeoxyglucose so the scan would reflect cerebral metabolism during the practice. Positron emission tomography (PET) imaging was performed approximately 30 minutes later.

**Results:** In the female participants, the meditation state showed significant decreases in the left inferior frontal, inferior parietal, insula, middle temporal, and orbitofrontal regions as well as in the right angular gyrus, anterior cingulate and parahippocampus compared to a neutral state (p<0.01). Male subjects had significant decreases in the left middle frontal, paracentral, precentral, and postcentral regions as well as the right middle frontal and paracentral regions during meditation (p<0.01). Men also had significantly increased metabolism in the cerebellum and right postcentral and superior temporal regions (p<0.01).

**Conclusions:** These findings represent a distinct pattern of brain activity, for both men and women, that is a hybrid between that of other meditation practices and sexual stimulation. Such findings have potential psychotherapeutic implications and may deepen our understanding of the relationship between spiritual and sexual experience.

## Introduction

Over the past 25 years, a variety of meditative and spiritual practices have been studied by our group and others with functional neuroimaging techniques. We have previously performed a number of functional neuroimaging scans as part of a larger program studying the effects of various meditative practices that have been previously published. ^1^^–^^5^ This research has led to a greater understanding of the brain structures and networks involved in various meditative practices. A number of structures appear to be associated with meditative and spiritual practices including the frontal and parietal lobes, and limbic structures, along with larger networks such as the default mode network (DMN) and the salience network. Other previous studies have shown how various meditation practices can affect brain processes related to emotion, cognition, and sensory experience. ^6^ However, additional research is required to further assess various types of meditative and spiritual practices, and their effects within the brain.

For the present study, we explored the neurophysiological effects of a unique meditation method called Orgasmic Meditation (OM). This practice involves female clitoral stimulation with a partner as a central focus (for this study a separate male partner was selected by each female subject as described in the methods section). An important point to consider initially is whether this practice can be appropriately classified as a form of meditation. For example, the definition of “meditation” given by Merriam-Webster’s dictionary is: “to engage in mental exercise (such as concentration on one's breathing or repetition of a mantra) for the purpose of reaching a heightened level of spiritual awareness”. While the definition on Wikipedia is: “Meditation is a practice where an individual uses a technique – such as mindfulness, or focusing the mind on a particular object, thought, or activity – to train attention and awareness, and achieve a mentally clear and emotionally calm and stable state”. Based upon the description of the practitioners, the OM practice appears to meet these basic definitional criteria by using a specific physiological process, in this case, clitoral stimulation, as a focus for the mind.

In a broader context, mindfulness is being attentive to what is – to the sensations in the body, to emotions, to thoughts – in the context of whatever the practitioner is doing. Thus, applying the principle of mindfulness to virtually any physical practice or process can turn that practice into a form of meditation. For example, walking meditation could be considered simply walking, breathing meditation simply breathing, and mindful eating simply eating. If one accepts that these other physical activities could be forms of meditation, then it is reasonable to conclude that a practice based upon what would normally be considered sexual stimulation can also be a form of meditation. Of course, there is certainly the potential concern that any effect of OM is more based on the sexual stimulation itself rather than on the meditative focus. However, there are traditional Hindu, Taoist, and Buddhist practices dating back thousands of years that use sexual stimulation in a similar manner. Ultimately, meditation is defined by those performing the practice, and any study that targets meditation has to assume that the practitioners are doing the specific practice and doing it the way they describe it.

According to OM practitioners, both the female and male participants are actively engaged in the practice and, hence, there is a specific effect to the male subject as well as the female subject. A recent study of the OM practice indicated that partners had improved health measures such as an increased sense of closeness. ^7^ Thus, it seems appropriate to explore the neurophysiological mechanism of action of the OM practice. One of the main advantages of studying this practice is that it is well characterized and can be performed for 15 minutes with an additional several minutes of preliminaries before starting and a brief concluding component (see below in methods for additional details). While this practice is called, OM, the goal is not specifically to achieve orgasm or climax but has the purpose of achieving a meditative state. For the purposes of this study, subjects performed the meditation in the same manner following the standard practice methods closely.

Since the overall goal of this study was to determine the neurophysiological correlates specifically during the practice, it was a challenge to determine the most appropriate technique for evaluating this practice. Furthermore, we were interested in observing neurophysiological changes in both the female and male subjects in order to determine the similarities and differences between them. Functional magnetic resonance imaging (fMRI) was not possible for studying the changes that occur during the practice since logistically, the two participants could not be in the scanner at the same time. In addition, the practice involves a moderate amount of motion that would adversely affect image acquisition, and hence, quality. We have previously used both positron emission tomography (PET) and single photon emission computed tomography (SPECT) to study meditation practices, particularly those that involve specific body positions or movements. ^8^ For the present study, we selected the use of ^18^F fluorodeoxyglucose (FDG) PET to specifically evaluate cerebral glucose metabolism. In order to perform this study, we placed an intravenous (IV) catheter into the arm of both the male and female participants prior to performing the OM practice. A bag of normal saline was connected to the IV and was placed on the other side of a screen to maximize privacy of the participants during the practice. We wanted to ensure that we were obtaining the cerebral metabolism associated not only when the subjects were clearly engaged in the practice, but also during the peak part of the practice. By injecting the subjects at the mid-point of the practice, we could effectively capture changes in cerebral glucose metabolism during the last half, or most intense part of the practice. We compared the cerebral glucose metabolism between the OM state and a “neutral” state. In the neutral state, the participants were located in the same room, positioned as they would be during the actual OM practice, and performing a sensory stimulation task by stroking the leg instead of the clitoris. Thus, the study was designed to match all aspects of the OM and neutral conditions with the exception of the clitoral stimulation during the meditation practice. The order of these two conditions were randomized and were done on two separate days. We should note that we previously published initial fMRI imaging data on the same participants as in this PET study. ^9^ Both the PET and MRI scans were performed on the same day, but as mentioned above, the MRI data were acquired immediately following the practice whereas the PET data acquisition was designed to help assess changes specifically during the practice. However, we found a number of significant changes in the brain regions of both the male and female participants (see below) and hence we hoped to compare those results to the changes in cerebral glucose metabolism observed during the OM practice.

Based on the current literature regarding meditation techniques, and the study design, we hypothesized that several brain regions would be particularly involved with OM. The regions that have been observed to be affected during other meditation studies include the limbic areas and insula associated with emotional processing, frontal regions and the anterior cingulate involved with attention, and posterior regions involved with the DMN (also see below). In our prior studies, in addition to those of others, we have found alterations in frontal lobe function during the practice of concentrative meditation techniques. ^1^^,^^2^^,^^10^ Specifically, activity in the frontal lobe is typically increased during practices that involve focused attention and decreased during practices that are associated with a sense of flow or sense of surrender. ^1^^,^^11^^,^^12^ Since the male subjects performing OM do report a subjective feeling of flow or “losing oneself” during the practice, we might expect reduced frontal lobe function. With regard to the female subjects, we might also expect a reduction in frontal lobe function since they are more passive as recipients of the stimulation, rather than performing an attentional task. The parietal lobe is another brain region affected by intense meditation practices typically showing decreased activity associated with subjects who describe a loss of the sense of self or a feeling of self-transcendence. ^1^^,^^13^^–^^15^ We predicted that there would be decreased parietal lobe activity associated with this meditation as well since practitioners describe similar experiences.

It is also important to consider potential brain changes associated more specifically with clitoral stimulation. Several PET and fMRI studies have explored the effect of manual clitoral stimulation, particularly during sexual orgasm, on the brain. The results have shown a blend of findings that depended on the specific results of the stimulation ( *e.g.*, for sensory reception, pleasure, orgasm). For example, a 15O H2O PET study found significant increases in cerebral blood flow in the sensory cortex and the inferior parietal lobe during clitoral stimulation compared to rest. ^16^ Proceeding to orgasm was associated with significant reductions in the regional cerebral blood flow (rCBF) of the left lateral orbitofrontal cortex, inferior temporal gyrus and anterior temporal pole. In both men and women, during sexual arousal, usually through visual stimulation, activation has been reported in several frontal lobe structures, as well as the thalamus, cingulate cortex, insula, and amygdala. ^17^^–^^20^ During orgasm in men and women, activation has further been reported in the cerebellum, anterior cingulate gyrus, and dopaminergic pathways. ^21^ This distinction between sexual arousal and actual orgasm or climax is important with respect to OM since the majority of times, such a climax is not attained. In fact, in our study, none of the women reported achieving actual climax during the practice.

Thus, we hypothesized that the practice of OM would appear to be neurophysiologically closer to meditation-based practices rather than sexual arousal or orgasm. However, given its approach, and the use of clitoral stimulation to enhance the meditative state, we hypothesized that the pattern of brain activity associated with OM would be unique and contain elements of both sexual stimulation and meditation, as well as elements that distinguish it from sexual experience and currently studied meditative practices such as mindfulness, loving-kindness meditation, or yoga. Therefore, we hypothesized that for women, there would be decreased activity in the parietal regions as well as the frontal lobe regions due to the meditative elements. We also might expect increased metabolism in the limbic areas, basal ganglia, and thalamus primarily due to sexual stimulation. In men, since there is no direct sexual stimulation at all, and participants report a subjective feeling similar to that of flow experiences, we expected that there would be decreased metabolism in the frontal lobe regions along with possibly increased metabolism in the limbic areas (due to some sexual arousal). We also expected that there might be alterations in the structures of the DMN including the parietal regions mentioned above and also the posterior cingulate cortex. Finally, since this is a paired practice, we expected the potential for changes in the social areas of the brain of both male and female participants, including the insula, angular gyrus, and supramarginal gyrus. The goal of the study was ascertaining whether the pattern of cerebral metabolism associated with this unique form of meditation was distinct in comparison to sexual stimulation alone and also other meditation-based practices.

## Methods

### Subjects

Subjects were recruited by the Marcus Institute of Integrative Health at Thomas Jefferson University Hospital, and data from the concomitantly acquired fMRI scans from these same subjects were previously published including information on their demographics. ^9^ Written informed consent, approved along with the protocol (IRB#11D.412; approved on 12 September 2019) by the Institutional Review Board of Thomas Jefferson University, was obtained from all subjects by the principal investigator prior to undergoing study procedures (typically on the day of the initial scanning). All procedures performed involving human participants were in accordance with the ethical standards of the institutional and/or national research committee and with the 1964 Helsinki declaration and its later amendments or comparable ethical standards. Blank consent forms, information sheets and case report forms can be found as *Underlying data.* ^22^

Subjects were healthy individuals who were included if they been performing the OM practice for at least one year on a regular basis (two to three times per month). Subjects were recruited between September 2019 and June 2020. During that time, all patients provided consent and underwent the various aspects of the study including PET imaging so that all data was collected during this period. A total of 20 female subjects were chosen first and they then selected their male partner who they had done the practice with before and who subsequently had to agree to participating. The number of 20 pairs was selected based on prior power calculations associated with changes in brain imaging associated with other meditation-based practices by our research group. Married individuals had to perform the practice with their spouse. Subjects were excluded by the principal investigator if they: (1) had any physical, psychological, or brain-related disorders that may affect cerebral metabolism; (2) were taking medications that could alter cerebral glucose metabolism; and (3) could not lie still in the scanner. Women of childbearing age were not included if they were pregnant or breastfeeding.

### FDG PET imaging protocol of meditation practice

The FDG PET was performed using standard of care imaging procedures at the Marcus Institute of Integrative Health. There were two test subjects for each session, a female subject and a male subject, who performed the OM practice as a pair in a closed room in the imaging facility. A physical screen was placed in the room for added privacy during the tracer injection. Both subjects had an IV catheter inserted prior to performing the practice and had an IV bag of saline placed on a moveable pole with an IV line connected to the catheter. The man was clothed throughout the practice and did not receive any direct sexual stimulation. The woman was clothed in a manner to allow her genitalia to be exposed during the practice. The room was prepared according to the standard practice methods. A blanket and pillows were placed on the floor to provide comfort and support during the practice. The female participants then laid down on the pillows with the male subject seated by her right side. The man performed stimulation of the clitoris for 15 minutes using a sterile glove and lubricant as needed. There was no risk of pregnancy or sexually transmitted diseases since there was no actual penetration or sexual intercourse. Prior to beginning the meditation, the two participants verbally informed each other that the practice is about to begin. They set a timer for 15 minutes and began the practice. A similar timer was also set by the research team. Halfway through the 15-minute practice, the principal investigator and research technologist quietly entered the room behind the screen in order to inject the tracer for the PET scans over a one-minute period, and then left the room so that the subjects could complete the practice with minimal disturbance.

For the PET scan, 148 to 296 MBq of FDG was injected *via* a manual bolus. When the practice was completed, the subjects rested for approximately 15 minutes and then were escorted into the scanner fully clothed. The female participant was scanned first, followed by the male subject, for every scanning session. PET and corresponding MRI images were simultaneously obtained on a 3T Siemens mMR PET-MRI scanner (Siemens Medical Solutions USA, Inc., Malvern, PA) over approximately 30 minutes.

A neutral, comparison state was also performed to account for the specific elements of the practice without clitoral stimulation component or the actual meditation process. Thus, subjects had the IV catheters and line placed, following which they entered the meditation room and were positioned in the same fashion as during the actual OM practice. Instead of stroking the clitoris, the male participant was asked to stroke the female participant’s upper leg to account for motor activity in the man and the sensory response in the woman. Each pair performed this neutral condition for the same 15-minute time period as the OM practice and were injected half way through. After the 15-minute neutral condition, the subjects rested for approximately 15 additional minutes and then were brought into the scanner. Importantly, we randomized the ordering of the meditation and neutral conditions so that half the pairs did the meditation first and half did the meditation second. Also, none of the participants reported achieving orgasm during the practice.

All PET/MRI acquisitions included a Dixon sequence used for the derivation of standard MR attenuation correction maps by separating water, fat, and bone signal and automatically applying the calculated attenuation correction. Image reconstruction was performed using a Poisson ordered-subsets expectation maximization algorithm with four iterations and 21 subsets. This image processing producing an image with a matrix size of 344 x 344 pixels and a voxel size of 1 x 1 x 2 mm.

It should be noted that we did not use arterial sampling to measure absolute cerebral glucose metabolism, but rather evaluated relative activity in the selected regions. There are two main reasons that this was not used for the current study. Practically, it would not have been feasible to perform arterial sampling while preserving the integrity of the OM practice. Physical access to the subjects and movement of the subjects during the practice would make such measurements impossible to acquire. Also, this is an approach required when it is expected that different states would be associated with absolute changes in cerebral glucose metabolism. We did not expect this for the current study. In part, this is not likely since the subjects function as their own control and the practice is expected to result in different patterns of cerebral metabolism that would require normalization to whole brain activity anyway, which is taken into account in our current analysis. Thus, we were able to successfully observe relative changes in brain structures within subjects and between the two conditions.

### FDG-PET image processing

The 18F-FDG PET brain images were pre-processed using SPM12 (Wellcome Department of Cognitive Neurology, Institute of Neurology, London, UK) running on MATLAB (RRID:SCR 001622) 2020b (MathWorks Inc., Sherborn, MA). The processing steps are as follows: 1) Segmentation of the anatomical (T1-weighted) MRI data into gray matter (GM), white matter (WM), and cerebrospinal fluid (CSF) tissue compartments and creation of a skull-stripped version of the original anatomical scan used for PET-MRI co-registration. 2) PET-MRI co-registration: Using rigid-body transformations the spatial orientation of the PET and MRI images from the same subject were aligned. 3) Partial volume effect correction (PVEc) of PET images: voxel-based correction was applied using the Müller-Gärtner (MG) method provided by the PETPVE12 toolbox. ^23^^–^^25^ 4) Glucose Intensity normalization: The whole brain (grey and white matter) PET signal was used as a reference region to standardize the regional PET signal to standard uptake value ratio (SUVR) allowing for the direct inter-subject comparison of preprocessed PET data. 5) Normalization: deformations produced from the SPM12 segmentation implemented in step 1 were applied to the PVEc PET images to transform the functional data into the standard Montreal Neurological Image (MNI) space. 6) Smoothing: A 6-mm full-width at half maximum (FWHM) Gaussian kernel was used to smooth the final PVEc PET images.

### Statistical analysis

Statistical analysis was performed using statistical module in the Data Processing & Analysis for Brain Imaging (DPABI V5.1_201201 (RRID:SCR_010501)) ^26^ running on MATLAB R2020b (The Math Works, Inc., Natick, MA, United States). Voxel-wise two-sample t-tests were conducted to obtain the brain metabolism differences between each two groups. The resulting statistical maps were corrected for multiple comparisons to a significant level of p < 0.01 using Gaussian Random Field (GRF) theory correction with 25 voxels as the minimum cluster size. The clusters showing significant group differences between the OM and neutral states were selected as regions of interest (ROIs) and the average of SUVR was extracted over the mask of each ROI. Pearson’s correlation analysis was conducted to estimate the correlation between the intensity of experience scores and SUVR measurements for the whole group and then for the just male and female subject groups separately. The Benjamini-Hochberg false discovery rate (FDR) based on the selected ROIs was used in post-hoc fashion to reduce the risk of committing a type I error. Finally, we performed an exploratory analysis to assess whether there might be any correlations (Pearson correlation) between changes in metabolism in brain areas between two subjects in a given pair. The latter correlations might provide an interesting focus for future studies of practices that engage subjects in pairs or larger groups to ascertain whether there is some concordance or “resonance” between the brain of people doing a practice together.

### Qualitative measures

We asked all subjects to rate the quality of their OM experience by answering the following questions, which were also presented in our earlier article ^9^: Q1: “How similar was the process you completed to what you have typically known Orgasmic Meditation to be? (on a scale from 0, not at all, to 10, identical/indistinguishable)”; Q2: “How intense was the practice you just had? (on a scale from 0, not at all intense, to 10, the most intense OM experience you ever had; 5 would be your average experience)”.

## Results

The demographic information for the participants are given in Table 1, ^22^ including mean age, experience, and the subjective scores that the participants provided regarding the intensity of the meditation experience. This was the same group for which we presented fMRI data in a previous report. ^9^ We asked subjects to provide a description of how they would characterize the subjective experience of the OM practice. Several common statements included from the women: “I feel connected to my partner, connected to myself, and a feeling of joy and love in the world”. Men also reported a sense of “flow and connectedness with the partner” as well as a “greater sense of connection with the world”. The male subjects also had a strong sense of tactile or sensory awareness with their partner through the stimulation process. Both men and women frequently described a feeling of oneness, relaxation and joy during the practice, as well as a sensation of “energy” or “electricity” throughout their body.

**Table 1.** Demographic information about the participant groups.

Variable	Female subjects (N=20)	Male subjects (N=20)
Age, years, mean ± SD	39.0±10.1	40.8±9.7
Years of experience, mean ± SD	7.8±3.4	5.5±1.9
Intensity of the experience, mean ± SD	6.1±1.2	7.1±1.5

For the entire group, the OM practice during the study was rated as a mean of 9.7±0.6 (out of 10) in comparison with their usual meditation experience. The intensity of the experience during the OM practice was rated as 6.6±1.4 (out of 10) for the entire group and the breakdown by men and women is given below.

**Figure 1.**
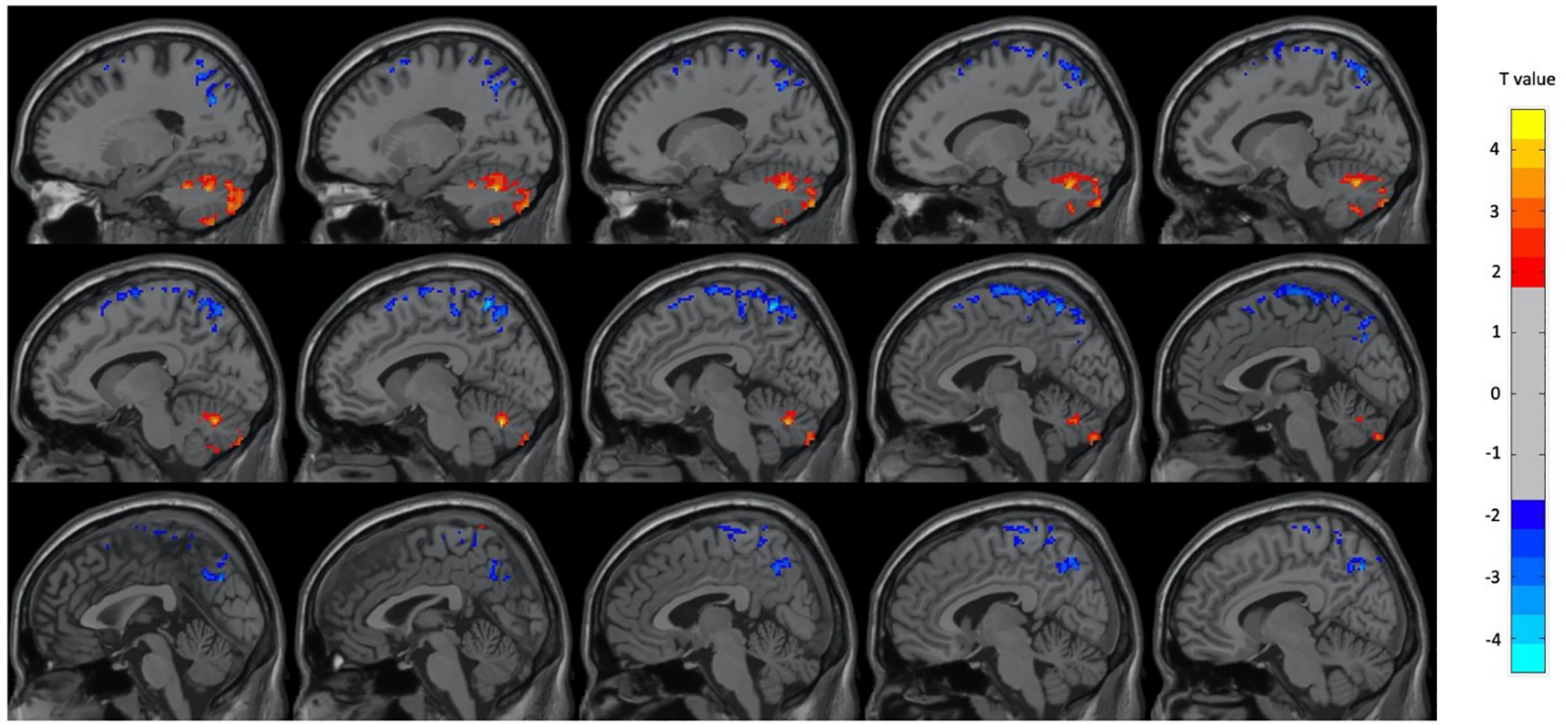
Select brain regions with significant differences between OM and neutral states in the total subject group. Two-sample t-test, p < 0.01, GRF-corrected, cluster size > 25 voxels. OM, Orgasmic Meditation; GRF, Gaussian Random Field.

**Figure 2.**
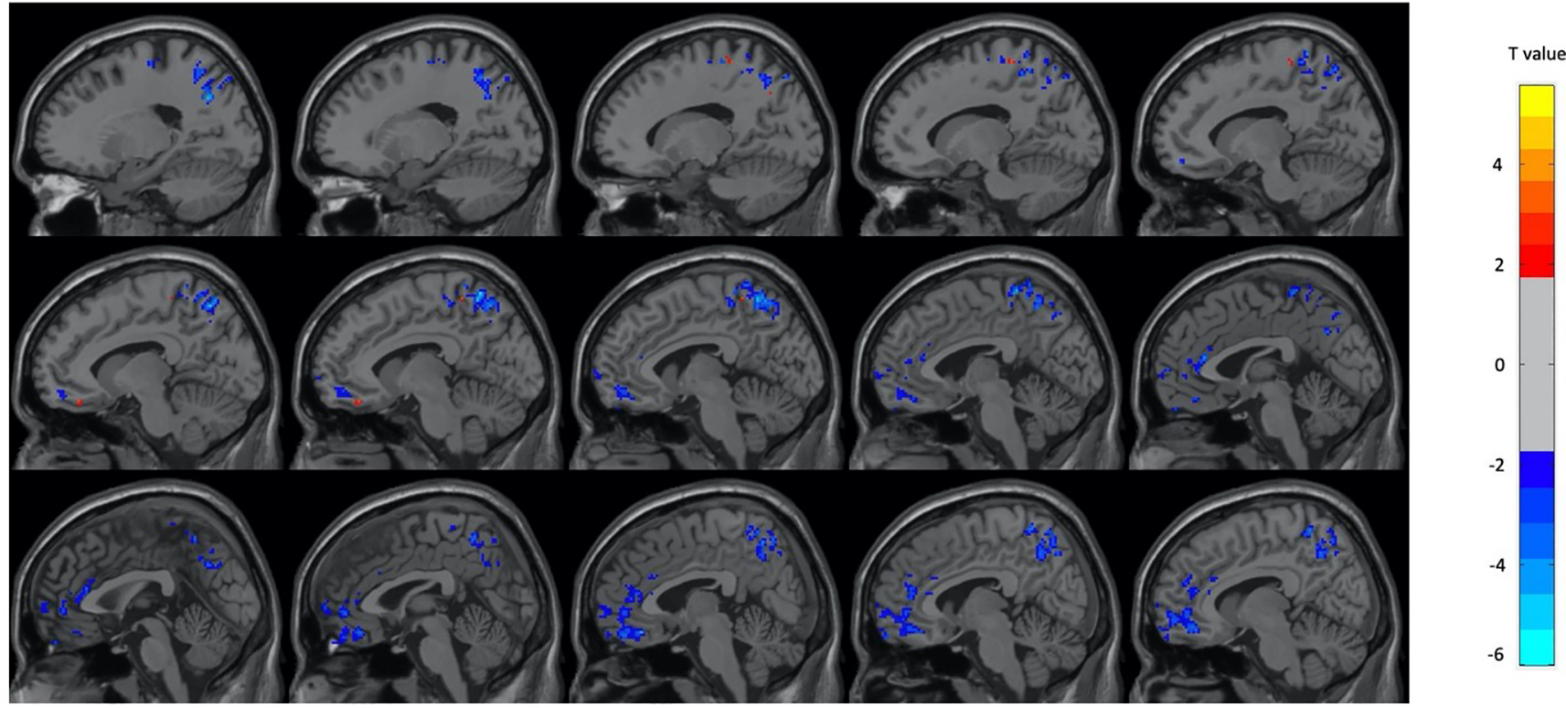
Select brain regions with significant differences between OM and neutral states in the female subject group. Two-sample t-test, p < 0.01, GRF-corrected, cluster size > 25 voxels. OM, Orgasmic Meditation; GRF, Gaussian Random Field.

**Figure 3.**
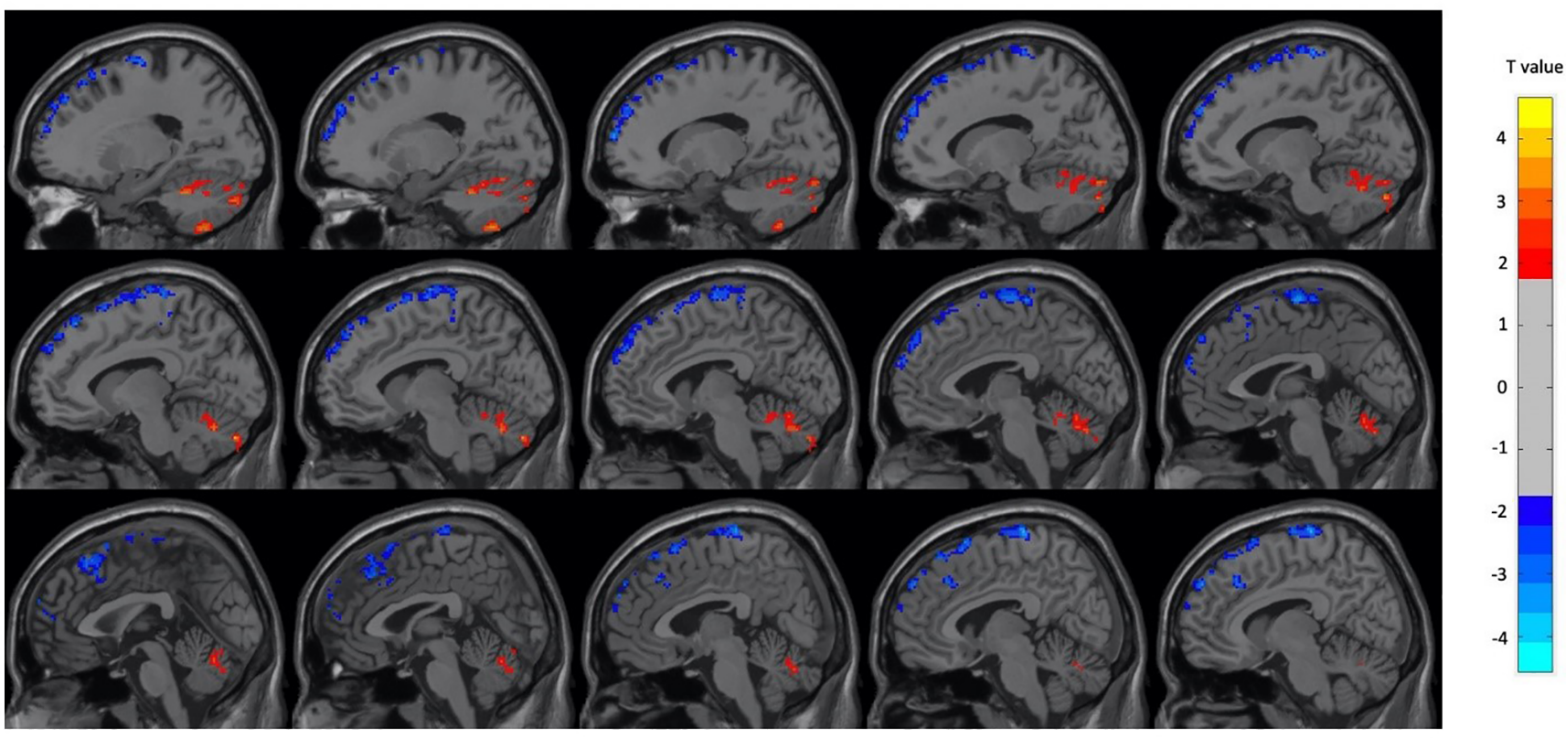
Select brain regions with significant differences between OM and neutral states in the male subject group. Two-sample t-test, p < 0.01, GRF-corrected, cluster size > 25 voxels. OM, Orgasmic Meditation; GRF, Gaussian Random Field.

Regarding the PET analysis, there were significant differences between the neutral and OM conditions for the group as a whole, and for the male and female subjects separately. Overall, there were many more regions with decreased metabolism during OM than increased metabolism. In the group as a whole, during OM there were mostly decreases in several frontal, temporal and parietal regions with increases only in the left cerebellum and right inferior and superior temporal lobes (p < 0.01, GRF corrected, cluster size > 25 voxels). The regions identified by the two-sample t-test are shown in Figure 1 and Table 2. In the female subjects only, there were decreases in metabolism during OM in several frontal, temporal, and parietal regions as well as the left insula, left precuneus, right angular gyrus, and right parahippocampus during OM. There was an increase in the right inferior frontal region only. The regions found to have significant changes in the female subject group are shown in Figure 2 and Table 3. In male participants, there were increases in the left and right cerebellum and right superior temporal gyrus during OM and decreases in several frontal, temporal and parietal regions. The regions found to have significant changes in the male subject group are shown in Figure 3 and Table 4. The unthresholded statistical maps between the meditation and neutral conditions can be accessed as a NeuroVault collection: https://identifiers.org/neurovault.collection:12567. ^27^

**Table 2.** Brain regions with significant differences between the neutral and OM states for the total subject group. Two-sample t-test, p < 0.01, GRF-corrected, cluster size > 25 voxels. The regions are based on AAL atlas. Negative peak intensity values reflect decreased metabolism during OM and positive values reflect increases. X, Y, Z coordinates of primary peak locations in the space of MNI. The voxel T threshold for voxel p threshold 0.01 is: 1.75. OM, Orgasmic Meditation; GRF, Gaussian Random Field; AAL, Automated Anatomical Labeling; MNI, Montreal Neurological Institute; T, statistical value of peak voxel.

Structure	Peak coordinate (X Y Z)	Peak intensity
Left Cerebellum	-26 -72 -52	3.14
Left Inferior Frontal	-42 24 26	-2.45
Left Insula	-34 22 0	-2.46
Left Paracentral Lobule	-4 -14 76	-2.44
Left Parieto-Occipital Region	-20 -62 36	-2.45
Left Precuneus	-16 -58 66	-2.45
Left Superior Parietal Lobe	-26 -66 54	-2.44
Right Anterior Cingulate	2 50 2	-2.46
Right Inferior Temporal Lobe	56 -36 -22	3.27
Right Postcentral Gyrus	34 -28 66	-2.47
Right Superior Temporal	62 -24 0	3.24

**Table 3.** Brain regions with significant differences between the neutral and OM states for the female subject group. Two-sample t-test, p < 0.01, GRF-corrected, cluster size > 25 voxels. The regions are based on AAL atlas. Negative peak intensity values reflect decreased metabolism during OM and positive values reflect increases. X, Y, Z coordinates of primary peak locations in the space of MNI. The voxel T threshold for voxel p threshold 0.01 is: 1.75. OM, Orgasmic Meditation; GRF, Gaussian Random Field; AAL, Automated Anatomical Labeling; MNI, Montreal Neurological Institute; T, statistical value of peak voxel.

Structure	Peak coordinate (X Y Z)	Peak intensity
Left Inferior Frontal	-42 18 22	-2.60
Left Inferior Parietal	-30 -56 46	-2.60
Left Insula	-42 -6 -4	-2.59
Left Middle Occipital	-28 -68 28	-2.59
Left Middle Temporal	-50 6 -26	-2.63
Left Orbitofrontal Cortex	-34 20 -14	-2.60
Left Precentral	-32 -16 48	-2.58
Left Precuneus	-10 -60 56	-2.58
Right Angular	42 -62 42	-2.60
Right Anterior Cingulate	14 36 18	-2.58
Right Inferior Frontal	46 28 4	5.60
Right Olfactory	24 10 -16	-2.59
Right Parahippocampus	40 8 -30	-2.60
Right Superior Parietal	26 -52 52	-2.59
Right Superior Temporal	44 16 -22	-2.59

**Table 4.** Brain regions with significant differences between the neutral and OM states for the male subject group. Two-sample t-test, p < 0.01, GRF-corrected, cluster size > 25 voxels. The regions are based on AAL atlas. Negative peak intensity values reflect decreased metabolism during OM and positive values reflect increases. X, Y, Z coordinates of primary peak locations in the space of MNI. The voxel T threshold for voxel p threshold 0.01 is: 1.75. OM, Orgasmic Meditation; GRF, Gaussian Random Field; AAL, Automated Anatomical Labeling; MNI, Montreal Neurological Institute; T, statistical value of peak voxel.

Structure	Peak coordinate (X Y Z)	Peak intensity
Left Cerebellum	-26 -72 -52	4.58
Left Middle Frontal	-44 18 52	-2.58
Left Paracentral Lobule	-4 -10 78	-2.58
Left Postcentral Lobule	-48 -10 62	-2.57
Left Precentral	-22 -12 70	-2.61
Left Superior Frontal	-24 42 40	-2.57
Right Cerebellum	38 -74 -38	4.24
Right Inferior Occipital	42 -78 -16	4.08
Right Middle Frontal	38 42 38	-2.58
Right Paracentral	10 -24 80	-2.57
Right Postcentral	44 -24 38	4.08
Right Superior Temporal	54 -38 22	4.09

In addition to these significant changes in brain function between the OM and neutral states, we found several significant correlations between changes in these specific brain structures and the intensity of the experience, which were distinct for the male and female participants. There were significant correlations in the female participants between the intensity of the experience and the change in metabolism in the left insula (r = +0.60, p=0.02) and right superior parietal lobe (r = -0.58, p=0.02). No correlations were observed between changes in brain structures and intensity of the experience in the male subjects.

Finally, we did an exploratory analysis to try to determine if there was any relationship between brain function within the pairs of participants. With this in mind, we found a number of significant correlations in the change of brain activity when directly comparing the individuals in each pair. In other words, activity changes in certain brain regions in the male subjects correlated with activity changes in certain brain regions in the concomitant female subjects. We present these findings as hypothesis generating data for potential future studies exploring practices that involve interactions between two or more subjects in which an analysis of the interacting individuals might be of value. The results are provided in Table 5. Notably, the intensity of the experience in the female participants correlated with the activation in several brain structures in the male participants, while the intensity of the experience in the male subjects correlated with the activation in several brain structures in the female subjects ( Table 6).

**Table 5.** Correlation analysis results between the change in specific region SUVRs in male *versus* female partners. The regions selected were those that were already found to have differences between the OM and neutral states. OM, Orgasmic Meditation; SUVR, standard uptake value ratio.

Female subject structure	Male subject structure	R correlation	Corr p value
Right Superior Temporal	Left Precuneus	-0.59	0.037
Left Insula	Left Precuneus	0.58	0.037
Left Parieto-Occipital	Left Superior Parietal	-0.52	0.041
Left Precuneus	Left Superior Parietal	-0.58	0.043
Left Insula	Right Postcentral	0.67	0.045
Right Superior Parietal	Right Superior Parietal	-0.54	0.045
Left Precuneus	Left Precuneus	-0.52	0.046

**Table 6.** Correlation analysis results between the intensity of the OM experience and the change in specific region SUVRs in male *versus* female partners. The regions selected were those that were already found to have differences between the OM and neutral states. OM, Orgasmic Meditation; SUVR, standard uptake value ratio.

Female subject structure/intensity	Male subject structure/intensity	R correlation	Corr p value
Intensity	Right Superior Temporal	-0.60	0.01
Intensity	Right Superior Parietal	-0.58	0.04
Intensity	Left Cerebellum	-0.58	0.02
Intensity	Right Precentral	0.57	0.03
Intensity	Left Insula	0.60	0.03
Intensity	Left Precuneus	0.76	0.02
Right Superior Temporal	Intensity	-0.60	0.04
Right Inferior Temporal	Intensity	0.56	0.03

## Discussion

To the best of our knowledge, this is the first study that has utilized FDG PET to evaluate the neurophysiological changes associated with OM, a practice that uses clitoral stimulation as a paired meditation practice between two individuals. It is important to emphasize that according to the practitioners, the practice is not designed to bring about sexual gratification, but to use clitoral stimulation to facilitate a meditative experience. Thus, the practice does not involve sexual intercourse, and the goal is not sexual climax or orgasm itself, but rather an intense meditative state. This is confirmed by their subjective descriptions of the experience, which do not use words related to eroticism or sexual arousal, but to feelings of awareness, connectedness, oneness, relaxation, energy, and joy. However, it is important to evaluate the neurophysiological mechanism of this practice and evaluate whether the findings resemble meditation, sexual stimulation, or a combination.

There are a number of aspects of this meditation practice that make it appropriate and interesting for scientific study. Importantly, it is a well-defined meditation practice that has clearly described elements, including enhancement of the meditative effect through the use of clitoral stimulation. The subjects interact in a similar manner each time regardless of the pair involved in the practice as well as how often the practice may be performed. The practice is specifically timed, which also makes it easier to study. Open ended, untimed practices such as mindfulness can sometimes be problematic for study since participants can meditate for several minutes to several hours.

Another noteworthy aspect of this practice is that it is performed as a pair. Participants have traditionally described that both individuals are engaging in the meditative practice, and while both participants describe the practice as a meditative state, because they are involved in distinct ways, we would hypothesize that male and female subjects would have differences in their subjective response and their physiology during the practice. It should be noted that while a female subject is always the recipient of the stimulation, the other participant can be male or female, although in our study, a male subject performed the stimulation of a female participant.

Given our team’s background in researching meditative and related spiritual practices, the opportunity to use neuroimaging to evaluate this unique practice can provide important information not only about the practice itself, but can be used for comparison with other practices. We hypothesized that the changes in brain function would be related in part to other meditative practices but could also have some similarities to clitoral or sexual stimulation/arousal. Given the approach and experiences that practitioners of OM report, we expected the pattern of brain activity to more likely represent a meditative experience rather than a sexual one. While we performed a whole brain voxel-based analysis, we hypothesized that specific brain regions, including the frontal lobe, anterior cingulate, temporal lobe, limbic regions, insula, basal ganglia, thalamus, precuneus, and parietal lobe, would be affected in a unique pattern of brain activity. We also hypothesized that there would be some similarities when the entire group is analyzed as a whole, and also patterns of activity distinguishing the male and female partners.

Our results are consistent with our general hypotheses regarding the physiological correlates of the OM practice. When the entire group was compared between the OM and neutral condition, a number of brain regions were found to be significantly different. As a whole group, there were significant decreases in the left insula, left inferior frontal gyrus, left paracentral lobule, left parieto-occipital region, left precuneus, right anterior cingulate, and right postcentral gyrus during the OM practice. There were significant increases in the left cerebellum, right inferior temporal lobe, and right superior temporal lobe.

However, as expected, there was a distinct pattern in the metabolic changes observed in the male *versus* female subjects. In the female participants, we observed multiple regions having significantly reduced metabolism. Specifically, there were significant decreases in the inferior parietal lobe, the precuneus, angular gyrus, and parahippocampus, which are all part of the DMN that has been shown to be decreased in other meditation practices. ^28^ These structures appear to be part of the medial temporal subsystem, which underlies spatial relationships utilizing auditory, visual, and somatosensory input. Furthermore, we have previously argued that decreases in the parietal lobe are associated with the subjective experience of a loss of the sense of self and a sense of connectedness that is common in meditative practices, including the OM practice. ^15^ Changes in parietal lobe activity have frequently been associated with other spiritual experiences such as self-transcendence. In addition, patients with lesions to the parietal lobe are more likely to express feelings of self-transcendence. ^14^ Male subjects also had mostly decreased metabolism in multiple brain regions, but with more frontal involvement, including the middle frontal, superior frontal, and precentral gyrus. There were also decreases in the paracentral and postcentral lobules. Increased metabolism in the male participants was observed in the cerebellum, superior temporal gyrus, postcentral gyrus, and occipital lobe.

The decreased metabolism observed in most of the brain structures during OM in both male and female participants is in contrast to sexual arousal during which studies have found activations in frontal lobe structures, the thalamus, cingulate cortex, insula, and amygdala. ^17^^,^^18^^,^^19^ Perhaps the study that most closely resembles the methods of the current study with regard to clitoral stimulation itself was performed by Georgiadis *et al.* ^16^ In this previous study, 12 female participants underwent clitoral stimulation to induce orgasm while undergoing ^15^H _2_O PET imaging to measure changes in cerebral blood flow. Our study is also similar in that, at least for the female participant, the stimulation was performed by a male partner. The results from the previous study observed sexual stimulation of the clitoris (compared to rest), which resulted in a significant increase in rCBF in the left secondary and right dorsal primary somatosensory cortex. In the present study exploring OM practice, we did not observe increased activity in these areas. In a study by Georgiadis *et al.*, compared with the control condition, climax was primarily associated with significantly decreased rCBF in the neocortex, specifically in the left lateral orbitofrontal cortex, inferior temporal gyrus and anterior temporal pole. These findings were not observed in the present OM study, but in the OM practice, climax is not specifically achieved. In male subjects, genital stimulation has been shown to produce activation in the midbrain, cerebellum, and dopaminergic areas, along with various cortical regions. ^29^^,^^30^ The current study of OM showed generally decreased metabolism, and it was not in the specific areas involved in sexual stimulation. In particular, we did not see significant increases in metabolism in the limbic areas, basal ganglia, or thalamus.

With regard to the specific comparison with sexual stimulation, several previous studies have found that climax in men and women is associated with activation of the cerebellum, anterior cingulate gyrus, hippocampus and amygdala, and the dopaminergic pathway structures. ^20^^,^^21^ During ejaculation in men, decreased activity has been reported in the temporal lobe and frontal lobe, alongside increased middle temporal gyrus activity. ^30^^,^^31^ Furthermore, activation in the orbitofrontal cortex has been reported during ejaculation. Activation of the frontal lobe has been observed in women during orgasm, but decreased activity in the frontal cortical regions has been observed during orgasm in men on PET studies and perfusion fMRI. ^32^ During OM, males in particular do not achieve climax or ejaculation. Furthermore, these patterns associated with sexual experience were not observed in either the male or female subjects performing OM.

As mentioned, in contrast to the women, men had decreased metabolism primarily in frontal lobe structures such as the inferior frontal gyrus. Such a finding has been observed in a variety of meditative practices and flow states and is consistent with what participants of the OM practice self-described as their typical experience. ^33^^,^^34^

Notably, men had increased metabolic activity primarily in the cerebellum. We have not specifically included the cerebellum in our analysis of meditation practices. There are a few studies that have implicated the cerebellum in meditation practices, especially focused based practices. ^35^^,^^36^ It is possible that the cerebellum plays a role in the DMN function. Recent studies have also shown the cerebellum is involved in emotional regulation, particularly negative emotions. ^37^ In the context of OM, it may be more related to the hand movements, which are specific and controlled during the practice. While this was supposed to be factored out in the neutral condition during which the male subjects made repetitive hand movements, but during OM, the movements are much more specific and closely monitored to stimulate the female effectively. Thus, future studies of OM and other meditation practices might help determine the role of the cerebellum in such practices.

A number of brain regions are implicated in different elements of meditation practices and are correlated with subjective experiences during meditation practices. For example, studies have shown frontal lobe activity to increase during attention focusing meditation practices. ^38^ Increased activity in the frontal lobe, and particularly the attentional network that includes the lateral prefrontal cortex, premotor cortex, lateral parietal regions, occipital regions, anterior cingulate cortex, and insula, has been observed during concentrative meditation techniques focusing on the breath or a mantra. ^39^ Other studies have linked long-term changes of meditation practice to changes in the precuneus and insula, along with fronto-parietal networks. ^40^ The insula in particular is an important structure with regard to emotional processing and perception. As with other meditation practices that evoke strong emotional changes or affect emotional regulation, the OM practice appears to be associated with altered insula activity supporting the notion that it functions as a meditation practice. ^41^ The precuneus is often linked to self-awareness and self-consciousness as part of its role in the DMN. ^42^ It has also been strongly linked to meditation practices and thus, is similarly affected during OM. In addition, other studies have shown that practices associated with a sense of flow and loss of purposeful control during meditation are associated with reduced frontal lobe activity. ^11^^,^^43^ We observed a similar decrease in frontal lobe function in the male subjects performing OM. This appears to be consistent with such flow experiences.

As mentioned above, several regions in the DMN were reduced during OM, which is consistent with findings from other meditation studies showing that the DMN becomes deactivated during a variety of meditative states. For example, Brewer *et al*., investigated the impact of several different types of meditation on the DMN. ^33^ The meditation practices studied included focused attention ( *i.e.*, concentration), open monitoring (choiceless awareness), and loving-kindness (a member of the constructive family) meditation. The DMN includes brain regions such as the medial prefrontal cortex, posterior cingulate cortex, precuneus, inferior parietal lobule, and inferolateral temporal cortex. ^44^ Brewer *et al.*, showed that the main nodes of the DMN were deactivated in experienced meditators. ^33^ The authors also reported a strong association between activity in the posterior cingulate, dorsal anterior cingulate, and dorsolateral prefrontal cortices. These findings are notable since these regions are involved in self-monitoring and cognitive control during meditation. Although some studies indicate that open monitoring meditation practices diminish DMN activity, ^45^ other studies have observed an increased activation of the precuneus during open monitoring in contrast to focused awareness practices. ^46^ In the current study, we observed a relatively small number of DMN structures affected during OM, suggesting a more specific effect of the OM practice on these structures. Mindfulness practices have also been found to be associated with altered activity in the salience, executive control, and orienting networks. ^47^ Again, in the present OM study, we observed changes in a number of these brain structures consistent with the mindful focus on the clitoral stimulation during the meditation practice.

To further evaluate some of the findings during the OM practice, we performed correlation analysis between changes in brain activity and the subjective intensity of the meditative experience reported by the participants. The female subjects showed a correlation between the change of activity in the left insula and right superior parietal lobe in comparison to the intensity of the experience. As we have mentioned above, these regions have been implicated primarily during various meditative practices, indicating that the OM practice represents a type of meditation. However, it is noteworthy that there were no correlations observed in the male participants. The reason for this lack of correlation between brain function and intensity is uncertain and future studies will have to try to better evaluate such relationships.

We also explored correlations between the male and female partners focusing on the structures that were already observed to be significantly affected during OM. While such an analysis is potentially problematic, especially because the men and women are engaged in different elements of the practice, we present these findings as hypothesis-generating data for future studies that may explore practices in which two or more individuals participate with each other. To begin, there were no significant correlations between the intensity of the experience in the male subjects compared to the intensity of the experience in the female subjects. This implies that while two members of the pair are engaged in the same practice, they do not necessarily have to have the same intensity of experience.

In terms of structures, there were significant correlations particularly between the left precuneus in both partners. Other regions that were also part of these correlations included the left insula, superior temporal gyrus, superior parietal gyrus. These correlations provide at least a potential hypothesis for future studies to explore how this practice, as well as other practices that involve two or more individuals, might result in reciprocal changes among the participants. These results imply that the activation of certain brain areas may have something to do with how the two participants engage in the mutual experience during the OM practice.

A primary limitation of the study centers on determining the best method for evaluating both participants during this practice. We selected FDG PET imaging, but theoretically, we could have considered SPECT imaging, which has tracers with a more rapid uptake into the brain. This might have allowed more precise capturing of the OM state. However, we expected that the majority of FDG was taken up during the practice time and any additional uptake occurred while at rest, which would theoretically diminish our sensitivity for observing significant findings. Thus, we feel that the findings are robust. The ordering of the two conditions (OM and neutral) were randomized so that the findings could not associated with increased comfort of the subjects with the laboratory environment or the imaging procedure. The study paradigm appears to have worked since we did not observe significant increases in the sensory or motor areas, which were accounted for by having similar components in both conditions. However, it is possible that some of the increases observed in the cerebellum were related to more coordinated movements during the practice. We did consider the possibility of having the participants perform clitoral stimulation in both the meditative and neutral conditions. However, the practitioners stated that it would be too difficult not to do the OM practice if they performed clitoral stimulation in the neutral condition. However, future studies might compare brain changes during the OM practice with other sexually stimulating states, including sexual climax, in addition to other meditation practices. Regarding the practitioners, many were highly experienced with the practice, performing it four to five times per day. However, it should be noted that since it is a 15-minute practice, four sessions per day would be one hour, an amount comparable to many other practices. In addition, it was noted that there was a substantial difference between male and female participants in terms of experience level. While it is certainly reasonable that the men and women can have different levels of experience, given that both groups had substantial experience, we achieved the stated goal of working with highly experienced individuals. Whether there are subsequent effects based on the duration or number of times performing the practice is an interesting question that can potentially be addressed in future studies with a larger sample size.

The findings of this study demonstrate specific patterns of metabolic activity associated with the OM practice that are unique for both the male and female participants. In addition, there are several brain areas that are significantly changed when considering both men and women together. These results are consistent with our initial hypotheses that there would be some similarities when comparing the entire group between the OM practice and the neutral condition. However, we also expected unique patterns of metabolic activity that differentiate the male and female participants. Overall, men tended to show a decrease in anterior structures, while women tended to show a decrease in posterior structures. Structures of the DMN and salience network were also involved.

The findings from this initial neuroimaging study of OM have potential implications regarding the psychological and neurophysiological processes involved in sexual stimulation and spiritual practices such as meditation. Future research can explore more specific neurophysiological correlates and compare the OM practice to other practices that include both meditative and/or augmentation components. In addition, studies can explore sexual experiences and the OM practice in the context of psychiatric disorders and their treatments, which are frequently associated with sexual problems.

Overall, these results demonstrate that the OM practice has unique characteristics that distinguish it from other meditative practices. Some of the brain changes are similar to those observed in concentrative meditation practices as well as mindfulness when it comes to changes in the frontal lobe and structures of the default mode and salience networks. There were a few brain changes similar to those involved with genital stimulation. However, the unique patterns observed with the OM practice indicates that it is a true hybrid practice.

## Data availability

### Underlying data

NeuroVault: OM Meditation Study. https://identifiers.org/neurovault.collection:12567. ^27^

Figshare: Orgasmic Meditation FDG PET Scans. https://doi.org/10.6084/m9.figshare.20113853. ^22^

This project contains the following underlying data:

- PET_AC.nii files (raw images from PET scans)
- OM Additional Data.2.xlsx (basic demographic information of subjects)
- 11D.412_fMRI PET OM Meditation comp_stamped version 12 10 2019 exp 09.09.2022.pdf (blank consent form and information sheet)
- fMRI PET MEDITATION CRF TC 2017_05_10 revised 2019_07_25.pdf (case report form)

Data are available under the terms of the Creative Commons Zero “No rights reserved” data waiver (CC0 1.0 Public domain dedication).
