# Supplementary material for: Alterations in cerebral glucose metabolism measured by FDG PET in subjects performing a meditation practice based on clitoral stimulation
Source: F1000Res. 2023 Dec 21;11:1015. Originally published 2022 Sep 7. [Version 2] doi: 10.12688/f1000research.122351.2 (PMC11809681; doi:10.12688/f1000research.122351.2)
Supplement: Peer review history

# Reviewer report: Reviewer response for version 2

**DOI:** 10.5256/f1000research.160302.r346500

**Published:** 2024-12-24

**Authors:** Abass Alavi

**Affiliations:** 1 Hospital of the University of Pennsylvania, Philadelphia, USA

These investigators (Dr. Andrew Newberg and his collaborators) have been pioneers in applying modern functional and molecular imaging techniques in assessing domains that have not been fully explored by other researchers. In particular, this group has published extensively on brain activation due to meditation in various settings with some excellent results. These articles have been published in major journals in the field of imaging and other disciplines over the past 3 decades. 

 Based on their past experience, these researchers have applied this approach to determine the relation between sexuality, or the libido, that have been debated in psychiatry. In this study, they report cerebral glucose metabolism in a unique mediation practice augmented by clitoral stimulation called Orgasmic Meditation where a spiritual state is noted to be attained by both male and female participants practicing the meditation as a pair. 

 Overall, this is very well-designed prospective research, which is unique in nature and further demonstrates the utility of PET technology in domains related to the overlap of both physiology and psychiatry. Therefore, publishing this paper will further strengthen the existing knowledge about using molecular and functional imaging techniques in various disciplines in medicine. As such, I would like to encourage the journal to index this paper as a novel scientific approach for future studies.

Is the work clearly and accurately presented and does it cite the current literature?

Yes

If applicable, is the statistical analysis and its interpretation appropriate?

Yes

Are all the source data underlying the results available to ensure full reproducibility?

Yes

Is the study design appropriate and is the work technically sound?

Yes

Are the conclusions drawn adequately supported by the results?

Yes

Are sufficient details of methods and analysis provided to allow replication by others?

Yes

Reviewer Expertise:

Molecular Imaging, particularly the use of Positron Emission Tomography (PET)

I confirm that I have read this submission and believe that I have an appropriate level of expertise to confirm that it is of an acceptable scientific standard.

# Reviewer report: Reviewer response for version 2

**DOI:** 10.5256/f1000research.160302.r232106

**Published:** 2024-05-06

**Authors:** Helen Lavretsky

**Affiliations:** 1 Department of Psychiatry, Semel Institute for Neuroscience and Behavior, University of California Los Angeles, Los Angeles, CA, USA

The authors have responded to my comments.

Is the work clearly and accurately presented and does it cite the current literature?

Yes

If applicable, is the statistical analysis and its interpretation appropriate?

Yes

Are all the source data underlying the results available to ensure full reproducibility?

Yes

Is the study design appropriate and is the work technically sound?

Yes

Are the conclusions drawn adequately supported by the results?

Yes

Are sufficient details of methods and analysis provided to allow replication by others?

Yes

Reviewer Expertise:

Integrative medicine, mind-body practices, biomarkers, neuroimaging

I confirm that I have read this submission and believe that I have an appropriate level of expertise to confirm that it is of an acceptable scientific standard.

# Reviewer report: Reviewer response for version 1

**DOI:** 10.5256/f1000research.134329.r188012

**Published:** 2023-11-24

**Authors:** Helen Lavretsky

**Affiliations:** 1 Department of Psychiatry, Semel Institute for Neuroscience and Behavior, University of California Los Angeles, Los Angeles, CA, USA

This study reports the findings from the PET study of the OM orgasmic meditation during clitoris stimulation in 20 women and 20 men partnering during this 15 min meditation compared to a neutral control of stroking upper leg.

 The results include regional reductions in brain glucose metabolism during stimulation compared to neutral state and some correlations between changes in men and women.

 This is really an interesting study and design, and an relatively unusual meditation with sexual stimulation.

 One could argue about control conditions- does stroking upper leg cause similar sensations?

 How about comparing it to meditation without clitoral stimulation? Would the satisfaction and brain results be similar or different.

 it is a nicely done observational study contributing to the literature of neural mechanisms of meditative experiences. Not sure what health benefits it might have other than mutual enjoyment by the partners.

 It would be nice to comment whether all couples were naïve to this type of meditation or true followers and "believers" than also addressing a "placebo" response in the the true followers of the practice.

Is the work clearly and accurately presented and does it cite the current literature?

Yes

If applicable, is the statistical analysis and its interpretation appropriate?

Yes

Are all the source data underlying the results available to ensure full reproducibility?

Yes

Is the study design appropriate and is the work technically sound?

Yes

Are the conclusions drawn adequately supported by the results?

Yes

Are sufficient details of methods and analysis provided to allow replication by others?

Yes

Reviewer Expertise:

Integrative medicine, mind-body practices, biomarkers, neuroimaging

I confirm that I have read this submission and believe that I have an appropriate level of expertise to confirm that it is of an acceptable scientific standard.

# Response

**Published:** 2023-12-16

**Authors:** Andrew Newberg

**Affiliations:** Thomas Jefferson University, USA

We greatly appreciate the reviewer's comments which we feel have helped to substantially improve the article. Below is a point-by-point response. Corresponding changes are made in the manuscript in track changes mode.

 This is really an interesting study and design, and an relatively unusual meditation with sexual stimulation. **Thank you for these positive comments.**

 One could argue about control conditions- does stroking upper leg cause similar sensations? **This is an interesting and important question. In planning for the study, we considered several options and decided that this would be the best as it matches movement by the male and the sensory reception of the female. However, it is not the same type of sensation since it is not a form of sexual stimulation. It might be interesting to compare clitoral stimulation with and without meditation, however, the practitioners were concerned that such a distinction might be difficult as it might overlap with their typical meditation. We have observed such an issue with our studies of other practices. For example, for people who meditate sitting still while focusing on their breath, they might start naturally meditating if placed in a room and asked to sit still and breathe normally. We have expanded on these issues in the manuscript.**

 How about comparing it to meditation without clitoral stimulation? Would the satisfaction and brain results be similar or different. **Yes, this would be another interesting comparison. We have expanded this discussion in the manuscript.**

 it is a nicely done observational study contributing to the literature of neural mechanisms of meditative experiences. Not sure what health benefits it might have other than mutual enjoyment by the partners. **Thank you for the positive comments. We hope that this data might be useful for informing future clinical studies that might focus on symptoms of depression or anxiety, as examples. We will expand upon possible future directions in the manuscript to address this comment and a similar comment by the other reviewer.**

 It would be nice to comment whether all couples were naïve to this type of meditation or true followers and "believers" than also addressing a "placebo" response in the true followers of the practice. **We agree that this is also an interesting issue. In order to perform this study successfully, we involved experienced practitioners. Future studies might evaluate whether people who are novices have similar effects and whether differences might be able to distinguish potential placebo effects.**

# Reviewer report: Reviewer response for version 1

**DOI:** 10.5256/f1000research.134329.r217436

**Published:** 2023-11-15

**Authors:** Michael Winterdahl

**Affiliations:** 1 Translational Neuropsychiatry Unit, Aarhus University, Aarhus, Denmark

Thank you for the opportunity to review the manuscript entitled 'Alterations in cerebral glucose metabolism measured by FDG PET in subjects performing a meditation practice based on clitoral stimulation'. This manuscript delves into the fascinating intersection of sexuality and spirituality, examining brain activity imaged with FDG PET during a unique practice of Orgasmic Meditation. The study involved 40 participants, comprising 20 males and 20 females, who engaged in a meditative practice enhanced with clitoral stimulation.

 Firstly, I would like to extend my congratulations to the authors. Securing funding and institutional approval for studies of this nature is a significant challenge, one with which I am personally familiar. The ability to conduct a sound and relatively well-powered PET imaging study under these circumstances is indeed impressive and commendable.

 Regarding the manuscript, I have a few concerns:

- The structure of the manuscript diverges significantly from the conventional format of introduction, methods, results, and discussion. Specifically, the introduction is overly lengthy, while the discussion is notably brief. It would benefit the reader if the introduction were condensed to provide only the necessary background and references to previous work and literature. Furthermore, in the discussion, the results should be contextualized within existing literature, including a thorough examination of the study's limitations. A balanced presentation of the study's strengths and weaknesses is essential (most have been mentioned in the introduction). Finally, the manuscript would be enhanced by suggestions for future research directions.
- In a previous paper, the authors published their findings from the same experiment using fMRI. However, they missed the opportunity to compare the fMRI findings with those from FDG PET, a very different imaging modality. The comparison of results between the two techniques could be a major strength of the present manuscript. It would have been beneficial to explore whether these findings correlate and whether the same regions are "activated".
- Since the subjects are not being stimulated in the PET scanner, is the argument for using FDG PET imaging really to avoid motion artefacts? Or does the main advantage lie in the biochemical information obtained? Can the authors discuss this further to clarify the details?
- Is the dopamine system involved in meditation? What would, e.g. Raclaprice PET have shown? Could other tracers have contributed with important knowledge - Can the authors discuss this further to clarify the details? 
- In a few instances, the participants or volunteers were mistakenly referred to as patients.
- Test values are typically denoted with a lowercase "t" (represented using a colour scale in figures).
- Although the population has been described previously, a small recap on the demographics could help the reader.

Despite the difficulty of the subject, the study was well conducted and, with a few necessary adjustments, absolutely publishable.

Is the work clearly and accurately presented and does it cite the current literature?

Partly

If applicable, is the statistical analysis and its interpretation appropriate?

Yes

Are all the source data underlying the results available to ensure full reproducibility?

Yes

Is the study design appropriate and is the work technically sound?

Yes

Are the conclusions drawn adequately supported by the results?

Yes

Are sufficient details of methods and analysis provided to allow replication by others?

Yes

Reviewer Expertise:

PET imaging, sexology, behaviour.

I confirm that I have read this submission and believe that I have an appropriate level of expertise to confirm that it is of an acceptable scientific standard, however I have significant reservations, as outlined above.

# Response

**Published:** 2023-12-16

**Authors:** Andrew Newberg

**Affiliations:** Thomas Jefferson University, USA

We greatly appreciate the reviewer's comments which we feel have helped to substantially improve the article. Below is a point-by-point response. Corresponding changes are made in the manuscript in track changes mode.

 Firstly, I would like to extend my congratulations to the authors. Securing funding and institutional approval for studies of this nature is a significant challenge, one with which I am personally familiar. The ability to conduct a sound and relatively well-powered PET imaging study under these circumstances is indeed impressive and commendable. **Thank you for these kind words. It is certainly a challenge performing such studies and we appreciate your comments about this difficulty.**

 Regarding the manuscript, I have a few concerns:

- The structure of the manuscript diverges significantly from the conventional format of introduction, methods, results, and discussion. Specifically, the introduction is overly lengthy, while the discussion is notably brief. It would benefit the reader if the introduction were condensed to provide only the necessary background and references to previous work and literature. Furthermore, in the discussion, the results should be contextualized within existing literature, including a thorough examination of the study's limitations. A balanced presentation of the study's strengths and weaknesses is essential (most have been mentioned in the introduction). Finally, the manuscript would be enhanced by suggestions for future research directions. **Thank you for these suggestions. We have modified the manuscript by shortening the Introduction and adding substantially to the Discussion section. We have also expanded a discussion of limitations and suggested future research directions. **
- In a previous paper, the authors published their findings from the same experiment using fMRI. However, they missed the opportunity to compare the fMRI findings with those from FDG PET, a very different imaging modality. The comparison of results between the two techniques could be a major strength of the present manuscript. It would have been beneficial to explore whether these findings correlate and whether the same regions are "activated". **We agree and have expanded our discussion regarding the connection of the current reported PET results with those from the MRI analysis and discussed its implications.**
- Since the subjects are not being stimulated in the PET scanner, is the argument for using FDG PET imaging really to avoid motion artefacts? Or does the main advantage lie in the biochemical information obtained? Can the authors discuss this further to clarify the details? **Thank you for this question. There were several factors that the PET imaging provided an advantage including the ability to perform the entire practice outside the scanner while still being able to assess metabolic changes in the brain during the practice. This includes accommodating the subjects to be able to be positioned and touching in the prescribed manner of the practice, reduction of motion artifact, and the ability to assess specific metabolic effects in the brain. We have clarified and expanded this in the manuscript.**
- Is the dopamine system involved in meditation? What would, e.g. Raclaprice PET have shown? Could other tracers have contributed with important knowledge - Can the authors discuss this further to clarify the details? **This is an important question. There is only one study we are aware of that used raclopride to assess a meditation state, but it would be quite interesting to observe changes in various neurotransmitter systems more specifically. We will include this in the Discussion section and also in future directions.**
- In a few instances, the participants or volunteers were mistakenly referred to as patients. **Thank you for pointing this out. They should be referred to as participants or subjects and we will correct this throughout the manuscript.**
- Test values are typically denoted with a lowercase "t" (represented using a colour scale in figures). **We agree and have modified this in the figure.**
- Although the population has been described previously, a small recap on the demographics could help the reader. **We agree and have expanded this discussion in the paper.**
